# Supplementary material for: IFN-κ directs antiviral immunity in human skin
Source: bioRxiv. 2026 Sep 23:2026.09.19.752886. Preprint. [Version 1] doi: 10.64898/2026.09.19.752886 (PMC13622050; doi:10.64898/2026.09.19.752886)

Figure S1. Antiviral transcripts are detected in healthy human skin cells. A) Violin plots showing expression of the ISGs *OAS1*, *MX2*, and *IFITM1* in keratinocyte, melanocyte, fibroblast, endothelial cell, and neuronal cell subtypes from healthy skin punch biopsies (GSE179633). Each dot represents a single cell. B) Violin plots showing expression of IFNλ subtypes in keratinocyte, melanocyte, fibroblast, endothelial cell, and neuronal cell subtypes from healthy skin punch biopsies (GSE179633). Each dot represents a single cell.

Figure S2. *IFNK*-regulates expression of canonical and non-canonical ISGs in keratinocytes. A and B) Volcano plots showing differentially expressed genes in A) sg*IFNK* #3 and B) sg*IFNK* #4 N/TERT2G keratinocytes compared to control (Cas9) cells. Dashed lines indicate differentially regulated genes with a cut off of -1.0 < log2FC >1.0 and padj < 0.05. C) Heat map of RNA-seq data showing all differentially expressed genes that are shared between sg*IFNK* #3 and sg*IFNK* #4 N/TERT2G keratinocytes compared to control (Cas9) cells. D and E) GSEA analysis of D) sg*IFNK*#3 and E) sg*IFNK* #4 N/TERT2G keratinocytes compared to control cells. F) Volcano plot showing differentially expressed genes from N/TERT2G cells treated with recombinant IFN-β (1 ng/ml) for 6 hrs. Dashed lines indicate differentially regulated genes with a cut off of -1.0 < log2FC >1.0 and padj < 0.05. G) Venn diagram showing overlap between recombinant IFN-β and IFN-κ regulated genes in N/TERT2G keratinocytes. H) Normalized counts for *MX2*, *OAS1*, *C3*, *LCP1*, *GALNT5*, and *GAMT* transcripts from RNA-seq data from control (Cas9) and sg*IFNK* #3 N/TERT2G cells. I) Normalized transcript counts for *MX2*, *OAS1*, *C3*, *LCP1*, *GALNT5*, and *GAMT* transcripts from RNA-seq data from N/TERT2G cells treated with recombinant IFN-β (1 ng/ml) for 6 hrs.

Figure S3. Validation of IFNAR1/2 and STAT1/2-deficient keratinocytes. A) Immunoblots of lysates from single cell clones (C#) from control (Cas9), *IFNAR1*-deficient, and *IFNAR2*-deficient N/TERT2G keratinocytes showing IFNAR1, IFNAR2, and IFN-κ protein abundance. Images are representative of n=3 biological replicates. B) Immunoblots of Cas9, *STAT1*-, and *STAT2*-deficient N/TERT2G keratinocytes showing STAT1, STAT2, and IFN-κ protein abundance. Images are representative of n=3 biological replicates.

Figure S4. Representative flow analysis of HSV-1 K26-GFP infected organoid fibroblasts

Gating strategy and representative flow plots used to identify GFP+ fibroblasts from HSV-1 K26-GFP-infected organoid cultures.

Figure S5. Extended data to support Figure 6. A and B) Immunoblots of lysates from Cas9 and *IFNAR1*-deficient fibroblasts treated with recombinant IFN-β or IFN-ɣ (100 pg/ml) for 15 minutes showing p-STAT1 (Y701) and total STAT1 protein abundance. C) Comparison of viral yields in Cas9, *STAT1*-, *STAT2*-, and *IRF9*-deficient fibroblasts infected with VSV (MOI 1.0) for 24 hrs and treated with IFN-β (100 pg/ml) at 1 hpi. Data are presented as the mean ± SEM of n=3 independent experiments. D) Heat map representation of RNA-seq data showing differentially expressed genes in Cas9 and *IFNAR1*-deficient HSE fibroblasts. DEseq2 was used to identify differentially expressed genes with a -1.0 < log_2FC >1.0 and padj < 0.05 cut off. E-G) *MX2* transcript abundance relative to *TBP* in DE and HSE organoid fibroblasts generated with G)*STAT1*-, F) *STAT2*-, and E) *IRF9*-deficient fibroblasts compared to Cas9 control cells. Data are presented as the mean ± SEM of n=3 independent experiments. H) qRT-PCR analysis of *MX2* transcript abundance relative to *TBP* in DE and HSE fibroblasts treated with 50 μM of Abrocitinib (JAK1i) or Deucravacitinib (TYK2i) for 24 hrs. Data are presented as the mean ± SEM of n=3 independent experiments.

Figure S6: Model figure for IFN-κ-mediated establishment of a pre-existing antiviral state in human skin

bioRxiv preprint doi: https://doi.org/10.64898/2026.09.19.752886; this version posted September 23, 2026. The copyright holder for this preprint (which was not certified by peer review) is the author/funder, who has granted bioRxiv a license to display the preprint in perpetuity. It is made available under a CC-BY 4.0 International license.

Figure S1. Antiviral transcripts are detected in healthy human skin cells

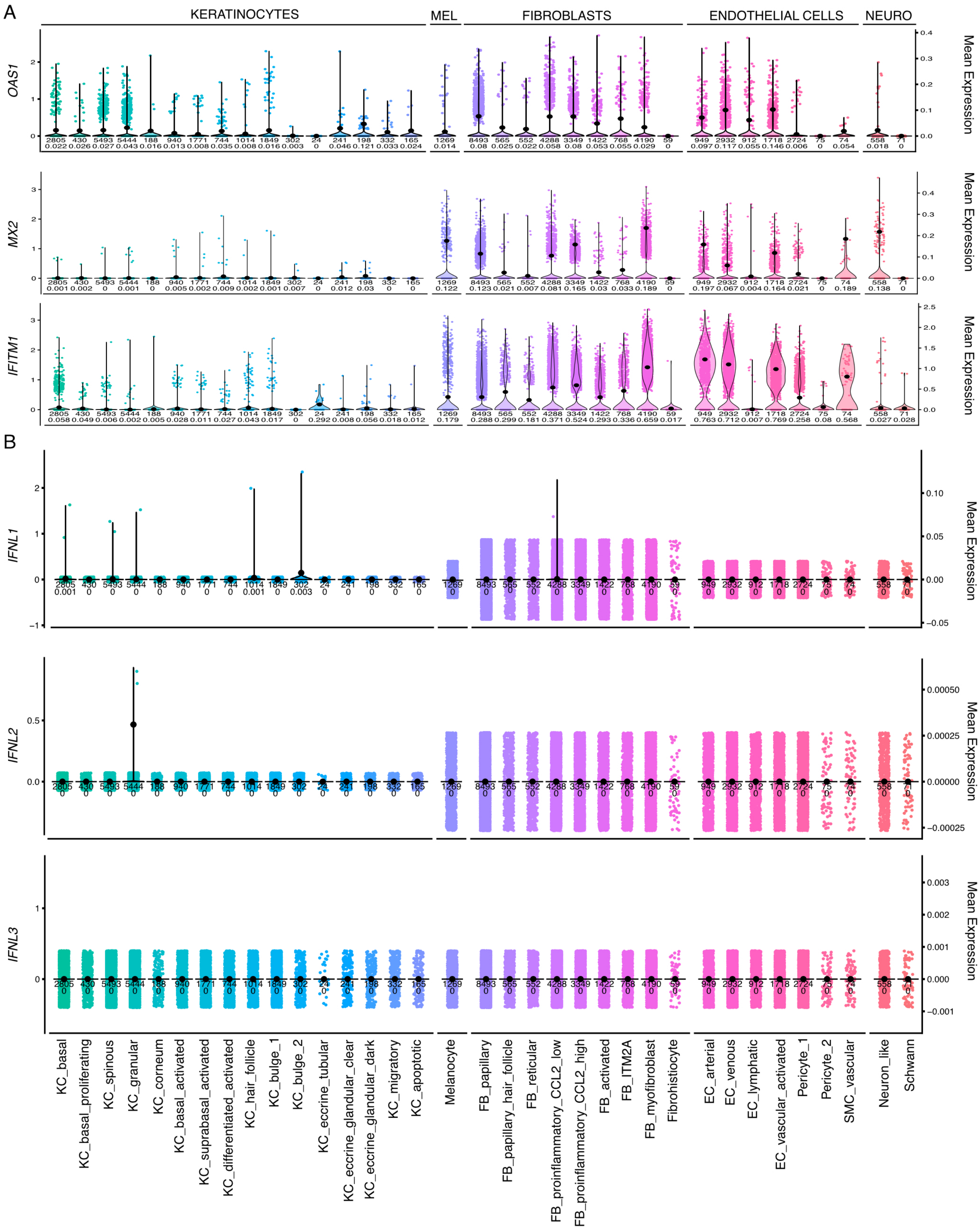

bioRxiv preprint doi: https://doi.org/10.64898/2026.09.19.752886; this version posted September 23, 2026. The copyright holder for this preprint (which was not certified by peer review) is the author/funder, who has granted bioRxiv a license to display the preprint in perpetuity. It is made available under a CC-BY 4.0 International license.

Figure S2. *IFNK*-regulates expression of canonical and non-canonical ISGs in keratinocytes

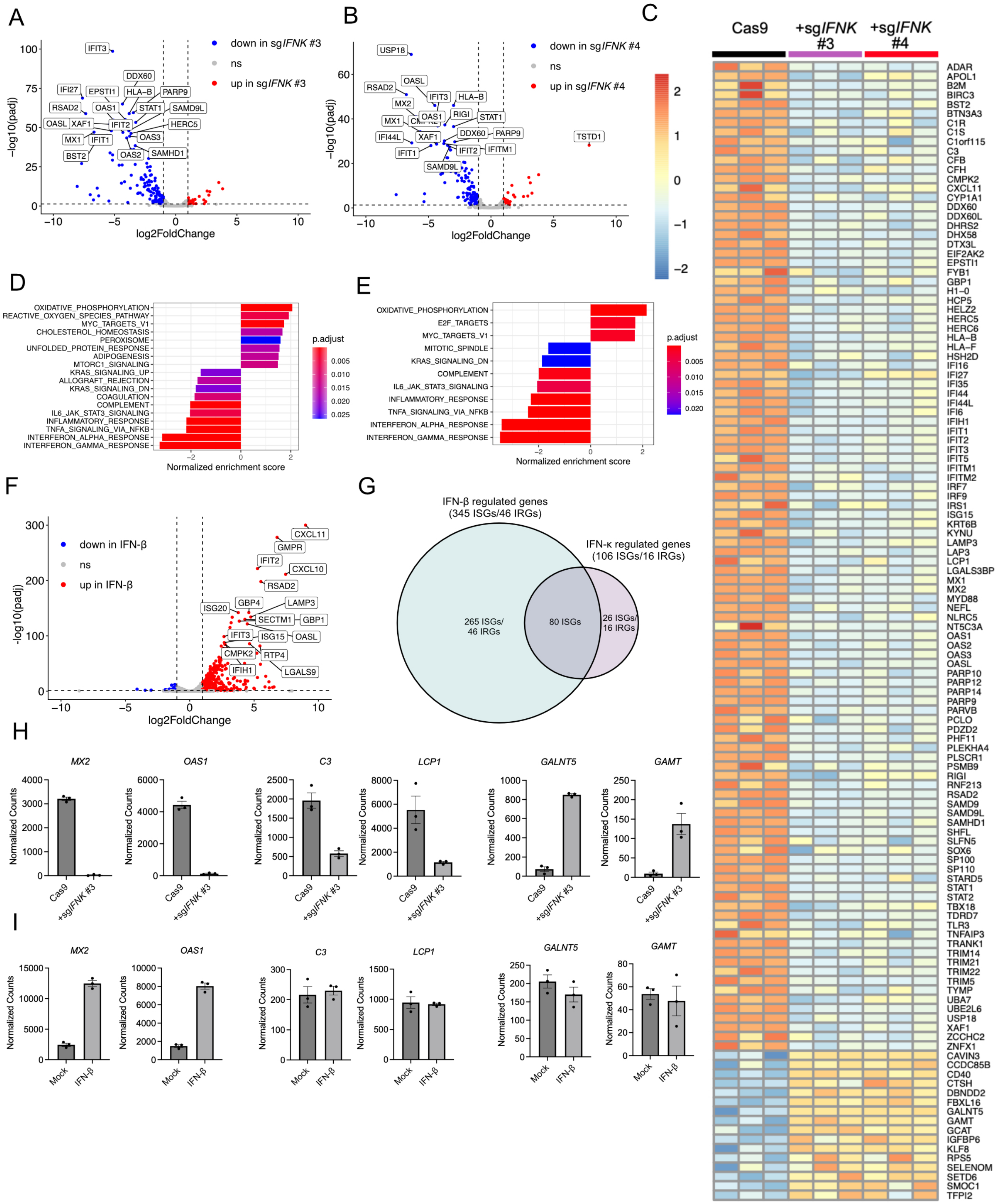

bioRxiv preprint doi: https://doi.org/10.64898/2026.09.19.752886; this version posted September 23, 2026. The copyright holder for this preprint (which was not certified by peer review) is the author/funder, who has granted bioRxiv a license to display the preprint in perpetuity. It is made available under a CC-BY 4.0 International license.

Figure S3. Validation of IFNAR1/2 and STAT1/2 -deficient keratinocytes

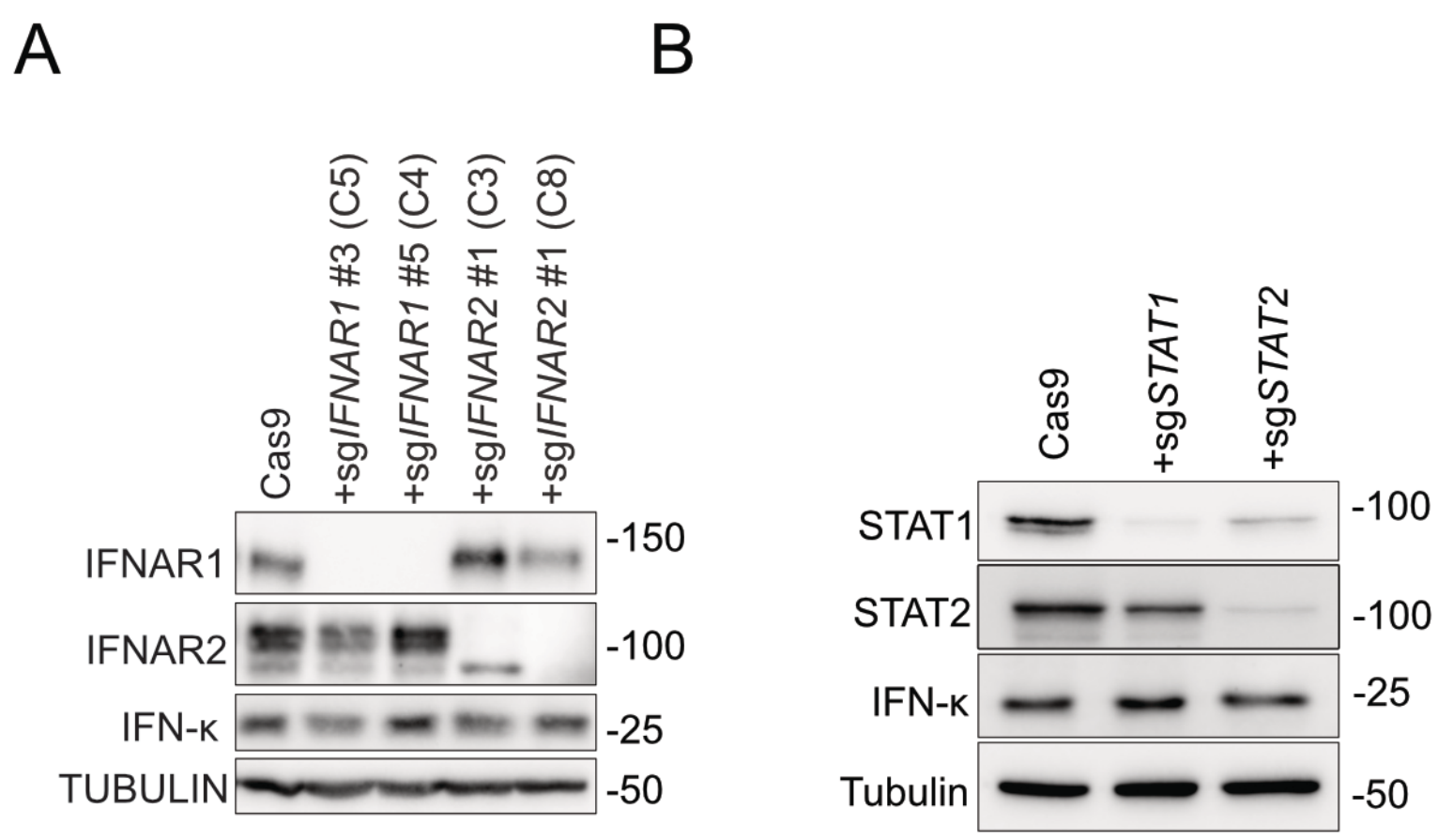

bioRxiv preprint doi: https://doi.org/10.64898/2026.09.19.752886; this version posted September 23, 2026. The copyright holder for this preprint (which was not certified by peer review) is the author/funder, who has granted bioRxiv a license to display the preprint in perpetuity. It is made available under a CC-BY 4.0 International license.

Figure S4

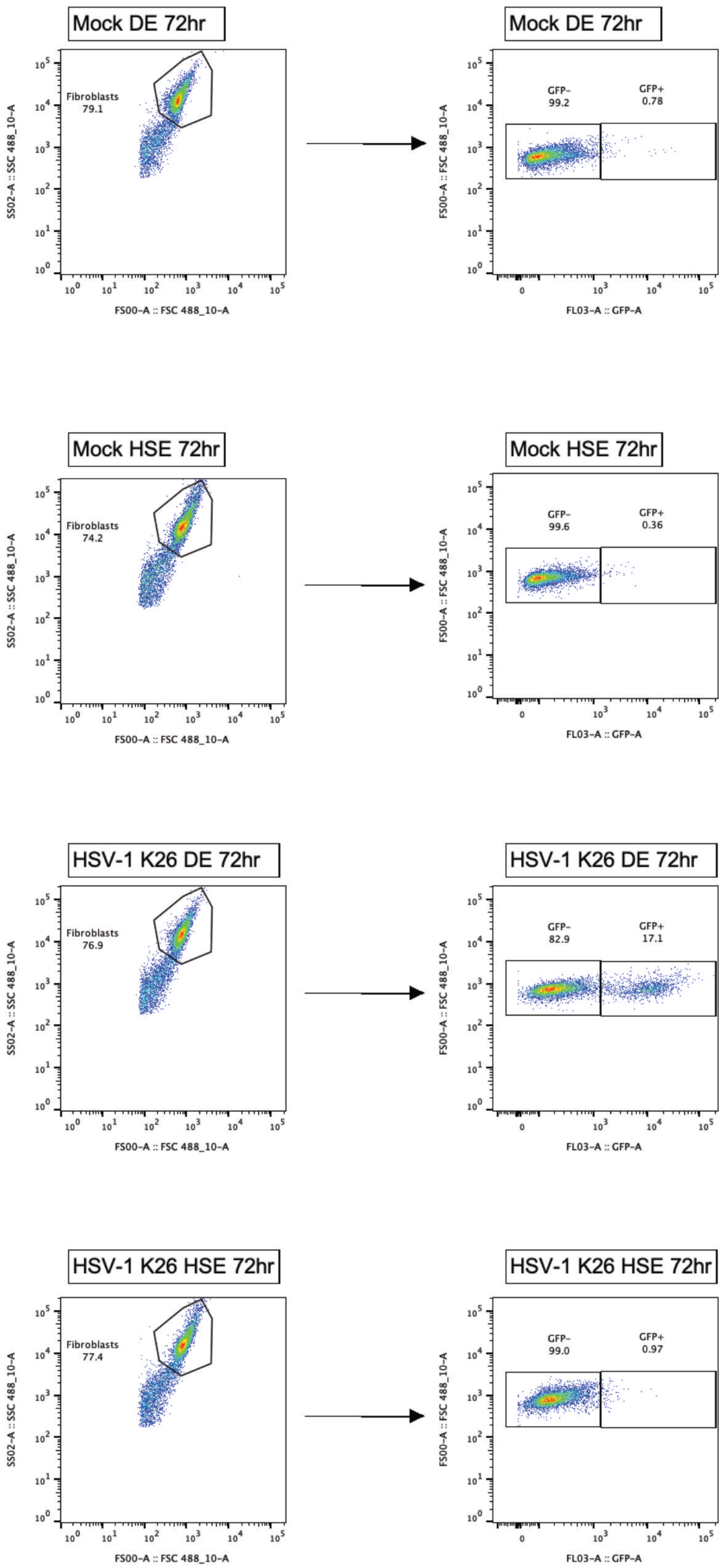

bioRxiv preprint doi: https://doi.org/10.64898/2026.09.19.752886; this version posted September 23, 2026. The copyright holder for this preprint (which was not certified by peer review) is the author/funder, who has granted bioRxiv a license to display the preprint in perpetuity. It is made available under a CC-BY 4.0 International license.

Figure S5

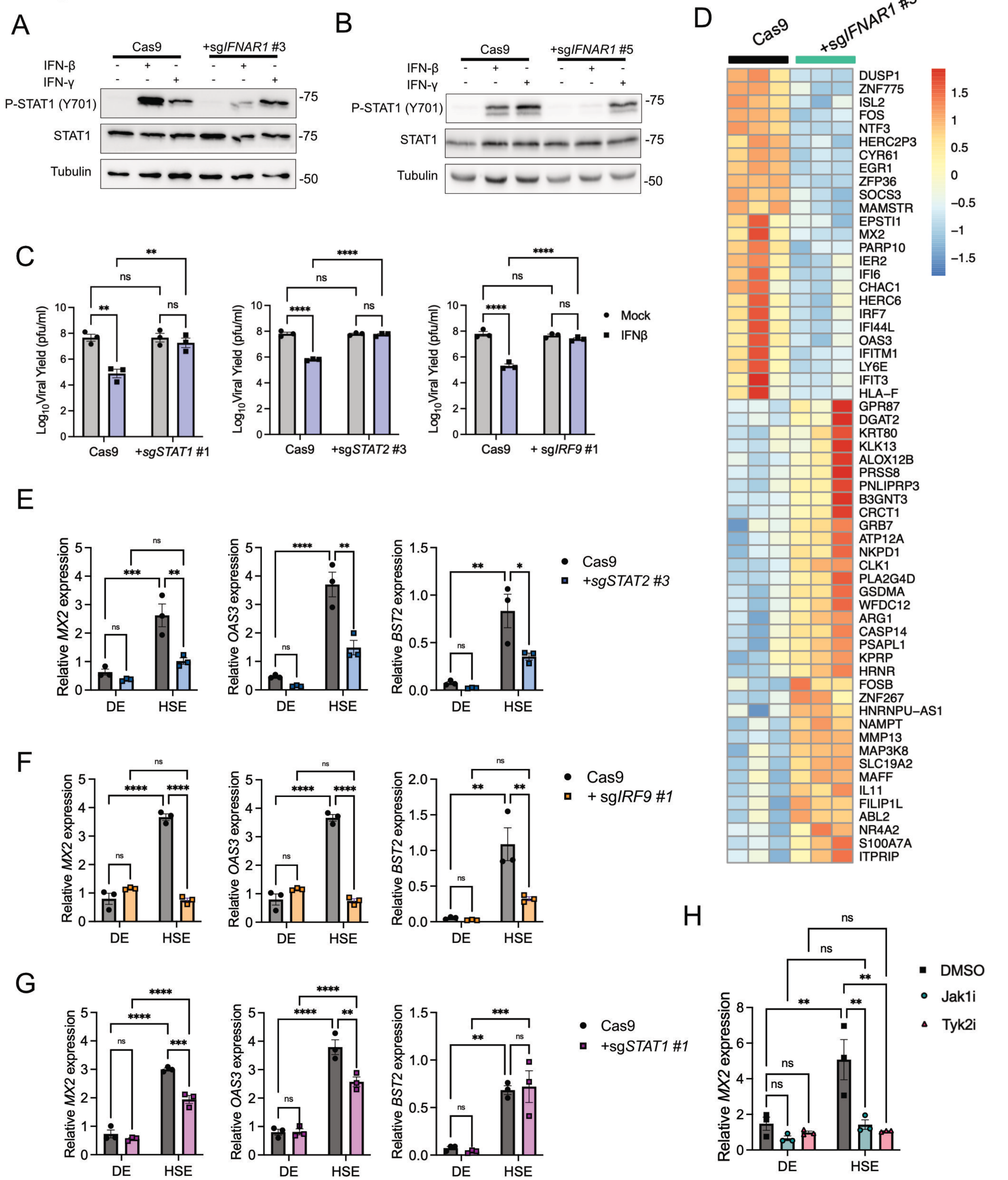

bioRxiv preprint doi: https://doi.org/10.64898/2026.09.19.752886; this version posted September 23, 2026. The copyright holder for this preprint (which was not certified by peer review) is the author/funder, who has granted bioRxiv a license to display the preprint in perpetuity. It is made available under a CC-BY 4.0 International license.

Figure S6 Model Figure

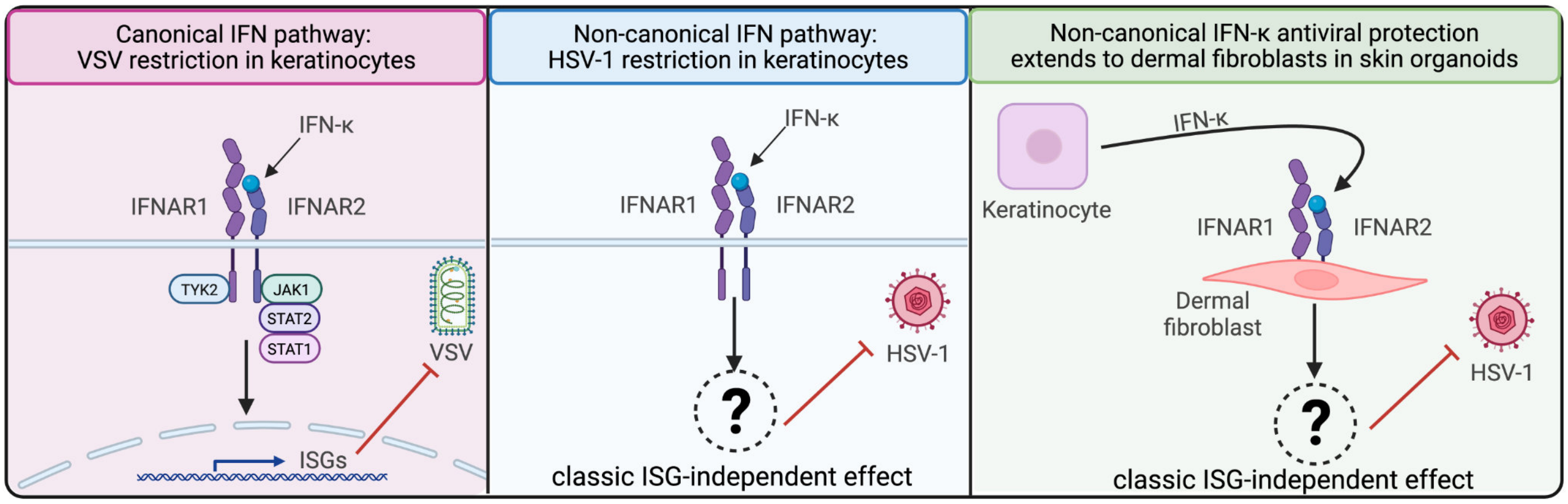

Supplement: Supplementary file 1 [file NIHPP2026.09.19.752886v1-supplement-1.pdf]
